# Comparison of governance approaches for the control of antimicrobial resistance: Analysis of three European countries

**DOI:** 10.1186/s13756-018-0321-5

**Published:** 2018-02-20

**Authors:** Gabriel Birgand, Enrique Castro-Sánchez, Sonja Hansen, Petra Gastmeier, Jean-Christophe Lucet, Ewan Ferlie, Alison Holmes, Raheelah Ahmad

**Affiliations:** 1 NIHR Health Protection Research Unit in Healthcare Associated Infection and Antimicrobial Resistance at Imperial College London, Hammersmith Campus, Du Cane Road, London, W12 0NN UK; 2 Institute of Hygiene and Environmental Health Charité, University Medicine Berlin Hindenburgdamm, 27D-12203 Berlin, Germany; 3 INSERM, IAME, UMR 1137, F-75018 Paris, France; 4 Univ Paris Diderot, Sorbonne Paris Cité, F-75018, Paris, France; 5 AP-HP, Hôpital Bichat – Claude Bernard, Infection Control Unit, F-75018 Paris, France; 6 Department of Management, King’s Business School, King’s College London, 30, Aldwych, London, UK

**Keywords:** Governance, Antimicrobial resistance, Infection control, Healthcare settings, Europe

## Abstract

Policy makers and governments are calling for coordination to address the crisis emerging from the ineffectiveness of current antibiotics and stagnated pipe-line of new ones – antimicrobial resistance (AMR). Wider contextual drivers and mechanisms are contributing to shifts in governance strategies in health care, but are national health system approaches aligned with strategies required to tackle antimicrobial resistance? This article provides an analysis of governance approaches within healthcare systems including: priority setting, performance monitoring and accountability for AMR prevention in three European countries: England, France and Germany. Advantages and unresolved issues from these different experiences are reported, concluding that mechanisms are needed to support partnerships between healthcare professionals and patients with democratized decision-making and accountability via collaboration. But along with this multi-stakeholder approach to governance, a balance between regulation and persuasion is needed.

## Background

The global challenge of antimicrobial resistance (AMR) requires coordination across governments, country borders, health and non-health sectors to maintain effectiveness of antimicrobials [1–3]. Effective governance has emerged as a crucial attribute deemed essential for sustained healthcare system performance and safety [4, 5]. To address AMR, effective governance needs to extend wider than considering just human health, including agriculture and animal health, but the coordination of efforts to manage inappropriate use of antibiotics within human health still requires much work [6–8]. Approaches to governance to address AMR serve as a test bed for health system governance more widely when thinking about specific and complex public health challenges.

The issue of AMR cuts across health conditions, organizations and professionals. It is estimated that around 700,000 people die each year from drug resistant infections and experts predict an alarming increase to 10 million lives each year by 2050 [2]. AMR threatens the practice of basic surgical procedures as well as advancements in medicine, as there are now high proportions of AMR in bacteria that cause common infections [6]. A key component of addressing AMR is to prevent health care associated infections; infections that develop as a direct result of medical or surgical treatment or contact in a healthcare setting [9].

The concept of health system governance in the health policy literature refers to "the processes, structures and organizational traditions that determine how power is exercised, how stakeholders have their say, how decisions are taken and how decision-makers are held to account" [10]. As defined by the World Health Organization, health sector governance refers to “a wide range of steering and rule-making related functions carried out by governments/decision makers as they seek to achieve national health policy objectives that are conducive to universal health coverage” [11]. This all-encompassing concept involves a complex mix of activities and political processes which collectively shape overall vision, strategy, and leadership approaches, intended to provide system wide assurance and stewardship [12]. In the last decade, a change in governance approaches is evident in many western healthcare systems. The shift in public perceptions from a position of full or assumed trust in healthcare professionals (HCPs) to a more critical stance has required increased transparency and accountability of individual and organizational performance [13]. In the context of healthcare-associated infections and antimicrobial resistance (AMR), such demands have been coupled with a reduced acceptance of events leading to harm [14]. But performance in infection prevention and control and antimicrobial prescribing across high, middle and low income countries remains sub-optimal. These societal changes, together with wider political and economic imperatives demand a rethink in terms of the governance approaches required to sustain effectiveness of antimicrobials [15, 16].

The inter-connected drivers of AMR and the complexity of patient pathways across and between community, primary and secondary care mean that no organisation in isolation can bring about the necessary impact. Regardless of national context, AMR is the result of a complex causal chain of structural factors and behaviours of the many individuals involved in patient care [17]. As locating individual practice which contributes to AMR is almost impossible, there has been a concerted shift in management practices towards shared responsibility between HCPs and patients [18]. In addition, policies have worked towards raising the profile of AMR, ensuring that prevention methods are evidence-based, and that systems of governance and accountability within healthcare organizations are sound [19].

At the individual country level, even within Europe, approaches to governance for the prevention of AMR vary in terms of the stakeholders involved and extent of regulation. Describing the landscape of healthcare governance and comparing strategies adopted by different countries can help to shape coordinated prevention and control of AMR at a global scale. This review therefore aims to examine some key theoretical concepts drawn from the public and health policy literatures to help us explore underpinning governance approaches for AMR prevention (emergence and spread) in healthcare across three European countries. By highlighting consistencies/divergences in such governance approaches, the aim is to accelerate learning at the country and also the regional level.

### A global threat requiring a re-look at governance approaches

As discussed above, the very nature of AMR as a policy issue which is associated with complex relationships across different industries and with various public agencies, as well as society at large, is the first driver for re-examining and if needed, updating governance approaches [1]. Strategies to address AMR may need to take account of these multiple sectors and players, and also be mindful of the wider consequences of the strategies adopted [8]. For example, the potential economic consequences of decreased productivity by removing antibiotic use in livestock must be taken into consideration when looking at the benefits of averting disability related loss of productivity by preventing infections in humans. A whole-systems perspective beyond health care is required, and within health care, ensuring strategy consistency across the whole health economy is needed too [2, 20].

Countries and actors (health and non-health providers) across each country are increasingly interdependent, acting as nodes of networks with multiple interactions. Social media and emerging technologies may have served, in essence, to help democratise engagement of citizens in governance and move it towards a co-production role by facilitating the whole-of-society (and not just experts) in creating and sharing knowledge and establishing relations between citizens, and citizens and experts, to maintain and preserve health (collaborative approach).

Having outlined our approach to analysing AMR as a health policy issue, we now describe our empirical methods. A documentary analysis for this paper was conducted by four researchers (GB, RA, EF and ECS) followed by expert input from AMR specialists (JCL, PG, SH and AH) for their knowledge of the international and national contexts. For each country, a documentary review of regulations/policies/guidelines and media coverage was conducted over the last 15 years using archival data of previous research conducted by the research team [20, 21]. Sources which enabled the authors to chart the trajectory of governance in the areas of IPC and AMR were accessed for data retrieval. Additional sources were included using a hand search and input of experts to update the existing database of sources from the published and grey literature. In total, 79 secondary sources were accessed. Expert input was also used to validate the emerging analysis using the analytical and theoretical framework described below.

### Conceptual framework of analysis: The health policy system and the AMR issue

The complex nature of system governance in this important AMR policy field may be helpfully analysed and understood by looking at (i) the overall health system level and (ii) the nature of a particular public health issue – in this case IPC and AMR, and indeed how these two levels of analysis interact. Differences in approaches within countries can be observed in this way as well as between countries. Our approach to analysis was informed by Smith et al. who seek to explore questions of the *how,* and *who* of three main governance processes, namely setting priorities, monitoring performance against these priorities, and accountability of all actors within the system for their expected contribution [5].

Our conceptual analysis was also informed by two main governance approaches observable in the public and health policy literatures (i) the exercise of top down power through hierarchy *or* (ii) more network governance approaches which include bottom up concepts of democratisation and collaboration, to provide insights into how the key characteristics of such policy systems may experience ‘evolution’ over time [22–24].

#### The traditional approach: Top down power through hierarchy

In many high income countries, the prevailing approach to AMR and infection prevention and control (IPC) governance remains characterised as hierarchical forms of power distribution and authority [25]. This hierarchical approach is based on a unique locus of control and directives (command) by high level authorities with standards backed by sanctions and rewards. Such top-down systems include elements of AMR surveillance as well as external mandates, and these may have acted as catalysts for change. Hierarchical monitoring can stimulate action by steering healthcare teams toward a shared objective which has been prioritised through (usually) external agencies. Willingness to accept suggested interventions with a new collective responsibility can help existing motivation for change through to progress [26]. Along with these positive aspects, in the case of AMR, this governance strategy can produce several side effects: tunnel vision by focusing all efforts on a single intervention (e.g. hand hygiene) rather than other equally valuable issues (e.g. antimicrobial stewardship); mistrust, tensions between the different actors [27]. Ironically, these actors can react to close surveillance by “gaming” the system, for example resulting in an under-notification of cases [28]. This hierarchical governance approach faces many challenges in addition to these, emanating from the wider societal context. Hierarchical approaches may be slow and limited in response to the fast pace of change from globalization, information dissemination (speed and methods), the role of the private sector and citizens’ expectations [19].

#### Democratisation and collaboration as an emergent governance model.

Partly in recognition of the limitations of top down approaches to public policy, some public policy authors [24] have explored what can be called new ‘network governance’ approaches. Within network governance, central government takes a less directive and more shaping role. There is more bottom up influence from civil society, Non-Governmental Organizations, active user groups and also public private collaborations.

Within the specific policy field of AMR, maintaining benefit to individual patients without compromising societal health and organizational viability (notably that of pharmaceutical companies) will require reframing solutions toward “shared value” approaches [18]. This objective requires (from a network governance prism) multi-level, multi-stakeholder approaches including government, private sector and civil society (including active bottom up influence from patient groups).

Public-private partnerships may contribute significantly to maintain the effectiveness of antimicrobials but will require reorientation of basic premises of system governance. As an example, in 2014, the European Union’s Innovative Medicines Initiative (IMI) launched a campaign to bring a complex mix of academic, biotech organisations and industry researchers together to work on the problem of AMR. This programme named “NewDrugs4BadBugs”, brings partners together to contribute not just for new molecular discoveries and antibiotic production, but also fast-tracking development in point of care testing for diagnosis, and at the same time looking for new business models of incentives for organisations and the industries involved. [29]. The programme has implemented an unprecedented sharing of knowledge, but equally must be supported by effective governance systems within and across industries and countries to assure the public interest is protected. .

New ways of healthcare governance now being adopted for assuring the quality of care and patient safety involve expert bodies and also user representatives [30]. This multi-stakeholder approach addresses the complexity of AMR through horizontal diffusion and decentralization of functions at all layers of the healthcare system and organizations within it. This evolution is toward a more democratic and collaborative process rather than authoritarian rules. It has the potential to change lines of accountability, increase information flows and involves users in new ways.

Given the potential scope and magnitude of impact of AMR on modern medicine and surgery and its high importance as a health policy issue, we therefore argue governance approaches could be driven in two alternative directions: either a move to tighten controls through the conventional hierarchical approach, or harnessing mixed models including market and collaborative network modes [5].

## Governance approaches within and across countries

### England

#### Overall health system governance

England has a centralized health system administered through the publicly funded National Health Service (NHS) (Table 1). In other parts of the UK (Scotland and Wales), health policy is a devolved matter, but the English regions have no such competence. Within England, *priorities* are centrally set by the Department of Health (DH), which sets out objectives for hospitals which are then operationalised through NHS England as a national managerial agency. Improvement campaigns are shaped by national institutions including the DH and local organizations (e.g. clinical commissioning groups). Public Health England (PHE, executive agency of the DH), supports local authorities in their duty to improve public health and reports annually on their progress. *Performance monitoring* is accomplished by centralised regulatory bodies (e.g. Care Quality Commission; NHS Improvement) National bodies (e.g. PHE) manage surveillance data and give advice on global or local trends and threats. In 2016, “NHS Improvement” was created as a powerful central regulator to take responsibility for overseeing foundation trusts and NHS trusts, as well as independent providers that provide NHS care, and hold providers to *account* for performance. Aims include the provision of support providers by giving information on their performance, reforming payment mechanisms and increasing patient involvement. In NHS foundation trusts, the board of directors is held to *account* by the council of governors composed of elected members (i.e. patient/user/carer, public and staff governors), and appointed key stakeholders [28]. NHS (Foundation) trusts are accountable to the Secretary of State for Health. Local authorities are now responsible for promoting public health of their local population. Recent policy has focused on ensuring market accountability up to the regulator with heavy top down control of the local financial deficits emerging in NHS Trusts. The system as a whole can be seen as generally top down in nature, with strong setting and monitoring of targets and performance indicators for local Trusts from the centre.

**Table 1:** Summary of health and IPC/AMR governance in the three included countries according to criteria defined by Smith et al [5]

		England	France	Germany
*Governance model*		• National Health Services (NHS): centrally planned health system • AMR: Hierarchical with authoritative pressure of DH	• Central-level governance model based on central government leading and setting directions for the health care system. • AMR: Hierarchical organization with authoritative pressure of the ministry of health and the Regional agency of health	• Federal government with corporate governance and the help of agencies • Wage-related contributions • 16 federal states (Länders) with their own administration • AMR: national and federal
*How priorities are set for improving actions and standards?*	*Who is involved & what is the role?*	• The DH & Care Quality Commission sets targets and puts in place the Outcomes Framework; Providing support, guidance, legislation, and Code of Practice. • NICE: provide clinical guidance.	• Ministry of Health via national agencies: Technical committee (High council of public health), policy group (Cosu Propias), the interministerial committee for health dedicated to AMR • Regional agency of Health: spell out criteria and targets for the provision of care.	• Bundesministerium für Gesundheit (BMG; Federal Ministry of Health) • The Commission of Hospital hygiene and Infection prevention (KRINKO) at the Robert Koch-Institute (RKI) • Possibility of local priority setting by federal states
	*What is the evidence base for decision-making?*	• Health technology assessment (rational arguments)	• Health technology assessment (rational arguments)	• Health technology assessment (rational arguments)
	*What are the main strengths*	• Transparency of information to public	• Performance management approach: Emphasis on structural and infrastructural aspects.	• Relatively strong degree of delegated and autonomous decision making.
	*What are the main weaknesses?*	• Difficulties to convert national goals into local practices • National targets led to local anomalies and unsustainable • Patient role not well defined. • Cost-effectiveness analysis studies not available	• Poor cost-effectiveness analysis	• Weak governmental powers. Decisions possibly blocked by nongovernmental and could delay the implementation of priorities • Risk of somewhat arbitrary goals by agencies.
*How is performance monitored?*	*By whom?*	• DH and PHE (NINSS): National surveillance. • NHS Improvement (formerly the Monitor): Intervene if concerns about performance of NHS foundation trusts. • Care Quality Commission: Inspections and assessments of NHS (foundation) trusts regarding national objectives.	• Ministry of Health: mandatory indicators with public reporting. • High Authority of Health (HAS): hospital certification. • Public Health of France and 5 interregional coordinating centres: Voluntary surveillance (RAISIN) for benchmarking.	• IQTIQ: Federal institute for quality management, quality report each year on federal level (formerly AQUA institute). • National Reference Centre for Surveillancce (Nationales Referenzzentrum für Surveillance von nosokomialen Infektionen, NRZ). Funded by the BMG, Its activities led to the creation of a national nosocomial infection surveillance system entitled Krankenhaus-Infektions-Surveillance System (KISS).
	*How and what are the main strengths and weaknesses?*	• Performance management approach: mandatory indicators with public reporting. Penalties and fines. • Empower patients. • Creation a culture of fearfulness and open up the possibilities of gaming. • Tunnel vision.	• Performance management approach: mandatory indicators with public reporting. • Tunnel vision.	• Mandatory for hospitals to survey nosocomial infections in high-risk areas (neonatal ICUs) and to record emerging multi-resistant nosocomial pathogens. • Nationwide surveillance of nosocomial infections, multi-resistant nosocomial pathogens and alcoholic hands rub consumption in Germany.
*How is accountability for performance ensured?*	*How are the accountability mechanisms in place linked to the health system’s broader governance structures?*	• Direct incentives through managerial control. • Financial pressure on contracts. • Public release of performance data, informed by goals and priorities, and serving a meaningful accountability process.	• Direct incentives through managerial control. • Public release of performance data, informed by goals and priorities, and serving a meaningful accountability process. • Financial penalties for not reporting data	• Statutory and voluntary accreditation schemes, at the organizational and practitioner level, and the freedom of patients to choose provider. • Confidential reporting of surveillance data
	*Are the mechanisms effective?*	• Increasing pressure for hospitals to produce and file plans for control activities with health authorities. • Increasing tendency for hospital and boards to be subject to audit. • Strong accountability structure in hospital trusts.	• No strong accountability structure.	• Weak governmental accountability.
To what extent are the three components aligned?		• Broad national goals must translate into achievable local targets. • Possible conflict between national and local priorities.	• Broad national goals must translate into achievable local targets. • Possible conflict between national and local priorities.	• Lack of capacity and coordination, technical difficulties. • Capture by powerful vested interests.

Abbreviations: *NHS* National Health Service, *AMR* antimicrobial resistance, *DH* Department of Health, *NICE* National Institute for Health and Care Excellence, *PHE* Public Health England, *NINSS* Nosocomial Infection National Surveillance System, Propias, Programme national de prévention des infections associées aux soins, *KRINKO* Commission of Hospital hygiene and Infection prevention, *RKI* Robert Koch-Institute, *IQWIQ* Federal institute for quality management, *AQUA* Institute for Applied Quality Improvement and Research in Health Care GmbH, *NRZ* Nationales Referenzzentrum für Surveillance von nosokomialen Infektionen, *BMG* Federal Ministry of Health - Bundesministerium für Gesundheit, *KISS* Krankenhaus-Infektions-Surveillance System, *ICU* intensive care unit

#### Governance for prevention of AMR in healthcare settings

The control of AMR is also highly centralised with national initiatives and commitments. *Priorities* for AMR prevention are set at the national level by the DH with a five year strategy [31]. Locally agreed ‘stretch’ targets (ie. to be achieved over an agreed timeframe requiring systemic improvements) for methicillin resistant *Staphylococcus aureus* (MRSA) (alongside national targets) were introduced in 2008 [32]. In 2013, the Chief Medical Officer’s annual report on the rise of AMR galvanized nation-wide focus [33]. In 2016, the government mandate to NHS 2016/17 clearly specified the need to support ambitions to prevent AMR [34]. *Performance* is monitored at the national level by mandatory surveillance. Efforts started in 2001 with the surveillance of MRSA first mandated as a core performance indicator for NHS trusts, followed by other blood stream infections including Glycopeptide-resistant *Enterococci* (GRE). In 2013 a “zero tolerance approach” to MRSA was initiated, with mandatory post infection review for each MRSA case [35]. The trajectory hence has been of increasing regulatory controls [20]. In 2014, PHE initiated the English surveillance programme for antimicrobial utilisation and resistance (ESPAUR) [36]. The structure of *accountability* is based on public reporting of national target indicators for individual hospitals. Financial penalties for MRSA cases were introduced in 2014 [37].

In 2016, PHE launched an open access portal of public health outcome indicators by geographic region and hospital (“fingertips” [38]) with AMR local indicators searchable down to hospital level. In 2017, this open access site incorporated primary care organisations, with antibiotic prescribing at general practice level available in the public domain for the first time. Reporting of antibiotic stewardship programme implementation at this more granular level is also set to follow. This demonstrates a move toward more comparative and transparent performance indicators and not just confined to hospitals.

### France

#### Overall health system governance

The French system is characterized by strong state regulation. In 2009, France initiated multi-level governance by empowering local regions to take a more integrated approach towards health financing and the delivery of care. *Priorities are established* at the national level by an assembly of experts gathered under the close supervision of the ministry of health (MoH). *Performance monitoring* is conducted by the MoH and national agencies such as Public Health France (PHF) created in 2016 to simplify and harmonise existing agencies. *Regulation, inspections and control* are routinely performed mainly at the regional level (Agence Régionale de Santé) and every four years by independent national health authority agencies (Haute Autorité de Santé, HAS), under the supervision of the MoH. In 2015, the health system was reformed significantly with the Health Act (Loi santé), centred on the whole patient pathway rather than a succession of interventions and providers, for collective *accountability* of financial and social consequences.

#### Governance for prevention of AMR in healthcare settings

*Priorities are set* at the national level by the MoH through a commission of experts. The policy is nationally driven by the PHF, and locally coordinated by regional authorities. In 2015, a national task force produced a report for the MoH giving a detailed timeline to reduce mortality due to AMR and of antibiotic consumption [39]. This report led to the creation of an intra-government committee dedicated to addressing AMR (across health/finance/agriculture/environment ministries). The recent national program (Propias) details specific targets for AMR prevention at national and local levels, orientated toward primary care and patient involvement [40]. *Performance is monitored* by a mandatory national notification system and nationally set indicators (alcohol-based hand rub consumption, antibiotic stewardship, and MRSA rates) under the supervision of PHF. A national network of voluntary reporting of multidrug-resistant organisms (MDROs) and antibiotic use (RAISIN) has been in operation since 1998. This is a confidential reporting and feedback system for benchmarking [41, 42]. The *accountability* function is performed by the public reporting of national indicators, available on the MoH website and newspapers. A financial penalty was introduced in 2010 for hospitals failing to publicise mandated indicators [43].

### Germany

#### Overall health system governance

In Germany, responsibility for the health system is divided between central government, the federal states and self-governing bodies. The Federal Joint Committee (G-BA) established in 2004 is the paramount *priority setting* body and takes responsibility for assessing the quality and efficiency of care. The German States (“Länder”) are responsible for the local implementation of G-BA priorities, with the jurisdiction to enact their own additional priorities. In 2009, the G-BA commissioned the AQUA Institute, an independent and impartial corporation, to implement nationwide cross-sectoral healthcare quality assurance. AQUA took responsibility for wide-scale *performance* reporting in an anonymized way, associated analysis, following up with underperforming providers. Such follow up includes request for a written report from hospitals to explain under performance (e.g., case-mix). If the AQUA is not satisfied with the report provided, the case is referred for external review to provide recommendations toward improvement. This whole procedure is called the “structured dialogue” (in German “Strukturierter Dialog”), suggesting norms of consensus building rather than top down directives. In 2014 the G-BA established the Institute for Applied Quality Improvement and Transparency in Health Care (IQTIG), an independent national agency contracted solely by the Federal Ministry of Health and the G-BA for the development of measures for quality assurance against which hospitals report. IQTIG is responsible for disseminating these quality reports, but the format and scope of this dissemination is yet to be agreed. It is not yet known when or how reports will be available in the public domain. According to IQTIQ´s specifications, hospitals are now required to publish various quality indicators in their annual mandatory quality report. *Accountability* is ensured through these various statutory and voluntary accreditation schemes, at the organizational and practitioner level, and the freedom of patients to choose provider.

### Governance for prevention of AMR in healthcare settings

At the Federal level, the key legal instruments for the surveillance, monitoring and prevention of AMR are defined in the Protection Against Infection Act (IfSG). The German States (“Länder”) have in some cases also issued supplementary regulations. Consistent implementation of legal targets by enforcement agencies, and by the responsible players in medical institutions, makes an essential contribution to achieving the central goal of the strategy. *Priorities* for AMR prevention are set at different levels, nationally with the German Antimicrobial Resistance Strategy (DART 2008 updated 2015) and regionally by networks created in 2004 with the support of the Robert Koch Institute [44]. National *performance monitoring* started in 2002 when the IfSG enacted mandatory surveillance by hospitals, of a defined set of MDROs [45]. Antibiotic resistance surveillance followed in 2007, collecting data on antibiotic resistance at the national level and informing antibiotic stewardship efforts [46]. In 2009, MRSA infections were mandatorily reported, followed by strains of Carbapenem-resistant *Enterobacteriacae* and *Acinetobacter spp.*(2016). In 2011, the IfSG expanded its duties with continuous monitoring, surveillance and interpretation of antibiotic consumption data in hospitals (supported by the newly created Federal commissions and surveillance systems managed by the Robert Koch Institute) [47, 48]. Since 1997, a voluntary and confidential national surveillance system (KISS) has been in operation for MRSA, GRE, gram negative MDRO, and use of alcohol-based hand rub [45]. *Accountability* is through the routine confidential reporting of surveillance data for participating hospitals, as described [49].

### Convergence and divergence of approaches across the countries

The three countries examined here all have formal mechanisms of setting comprehensive and clear goals for the health system. England and France have a vertical approach by setting priorities at the national level by the MoH with the advice of expert commissions. Policies are then nationally driven by agencies.

The health and AMR policy trajectory in England has been context specific and somewhat insular (even from the other UK countries); some local pressures have included the media, public and other political sources which have further legitimised a hierarchical governance approach [20] to governance in what is a highly centralised and managerialized policy system.

Some cross-national translation is seen in the recent creation of the PHF in France modelled on England’s independent institution, PHE. France has however, recently moved away from a top-down authoritative approach, to softer, multi-level governance, through empowering regions to take a more integrated approach in the financing and delivery of care. In Germany, the Federal states are historically empowered to set local priorities in addition to national ones. These devolved systems in France and Germany however bring different risks, as local states, counties or cantons have the freedom to depart from national priorities limiting comparability. Differences in goal setting and preventive strategies across localities can result in the spread of MDROs across regions (e.g. Länders) and countries [50]. However, a decentralised governance approach may provide possibilities for better monitoring of antibiotic usage through creation of a dedicated structure to assess and monitor governance at these local levels which is aligned with national, and international goals. The three countries in the present review appear heterogeneous in their strategies of AMR prevention. A better coordinated approach across countries may be beneficial. But at the same time relevance to context and history is important when suggesting new initiatives, for example public reporting was implemented in England 3 and 5 years ahead of other European countries and very much the norm; but is met with trepidation in less hierarchical health systems (eg. Germany, Switzerland), demonstrating the divergent contexts and potential work to be done if policy makers attempt to align approaches across nations with very different governance systems in health policy.

Common across the counties is a system of agencies advising the MoH to set high-level goals and choose national priorities. The three countries have also taken a similar route for *performance monitoring* but with different timelines for mandatory and voluntary surveillance and a different mix of indicators. In terms of *accountability*, England and France appear to place most reliance on transparent approaches open to public scrutiny, and regulated by information and benchmarking. Germany carefully promotes market instruments but as a corporatist healthcare system, the state takes a more passive role as a regulator through legislation and defining a framework – but providers and sickness funds act autonomously [51, 52]. These collectives are thus delegated responsibilities and achieve consensus through common need and objectives (eg. providers, insurance funds). However, a shift toward centralised public reporting is imminent following the establishment of comparable performance metrics. Observations arising from the analysis above may inform the future direction for AMR prevention and implications for health systems governance more generally.

### Network governance

In 2013, the Science Ministers of the G8 agreed that AMR demands an urgent global cross-sectoral response and research considering animal and human health, economy, industry, and the environment to accelerate improvements [53]. This ‘one health’ approach requires the development of collaborative approaches to governance; specifically through network governance [16, 54]. Yet our analysis shows that even within the healthcare context, a hierarchical approach dominates and a misalignment within country between the key dimensions of governance; priority setting, performance monitoring and accountability. Network governance requires shared ownership of goals, but flexibility of process for sustained improvement. This governance approach suggests that working together increasingly consists of functioning within complex networks rather than between clear hierarchical systems. It also implies bridging diverse policy areas, professional fields, academic disciplines, levels of governance (localities, states, regions and global) and sectors of society (public, private and civil). This approach was recently (2016) suggested in ‘The Review on AMR’ (often referred to as the “O’Neill Report” – after the economist who was commissioned to lead this review by the UK Prime Minister in 2014) advocating a supra-national entity to set global priorities, monitor performance and accountability [2]. Collaborative governance can however fail when there are conflicts between short term objectives, weak accountability, and differing power relations amongst different professional groups [55] (Table 2).

**Table 2:** Drivers and mechanisms for revising future governance for AMR prevention

Drivers for governance changes for AMR prevention	Mechanism for change	New perspective on AMR prevention governance	Objective	Suggested actions towards the new perspective governance for AMR prevention
Complexity^a^	Diffusion^d^	Network governance	Create a new academic/industrial/biotech skills mix based on systems thinking and complexity science.	Build inter-sectoral training in cooperation with schools of infectious disease, microbiology/hygiene, public health, business schools and policy.
			Increased accountability across healthcare and non-healthcare organizations.	Include HCPs, users, citizens and mass media in the governance approach and decision-making via independent agencies or organizations to implement new assessments and accountability frameworks.
	Diffusion and shared value^e^		Include AMR as a goal at governmental and societal level, as a key component of sustainable development	Engage organizations far beyond those involved in AMR sectors or even health (other ministers such as finance minister, business leaders, users) [2]
Inter-dependence^b^	Diffusion	Multi-level governance	Control interactions and promote coherence between sectors by an alignment of priorities	Cooperation among the various levels, e.g. geographical (regions and countries), clinical (primary and secondary care), species (antibiotics in humans/animals/agriculture/wider environment) following the “One Health” concept [61].
			Sharing information and experience	Pool reports of best and failed innovative practices in working with other organizations, groups, sectors via regular meetings for shared goals at the regional, national level and beyond.
		Mixing regulation and persuasion	Promote growing interest in nudge policies^f^	Monitoring progress through a mixture of hard and soft governance mechanisms to engage for AMR.
Co-production^c^	Diffusion	Adaptive governance	Transparency	Users, public and private organizations must work together to define new indicators for monitoring change and progress in AMR with new measures, shared for all parties and accessible to the public.
			Flexible and adaptable systems approaches with self-organization and decentralized decision	Create a dedicated structure to assess and monitor governance at the local, national, and international level, to adapt governance mechanisms to innovations (i.e. new mode of communication). Cybernetic approach^g^ utilizing communications and information for guidance and control.
	Democracy	Participatory governance, e-governance	Empower and involve users; public accountability; and strengthen health literacy	Dialogue with HCPs, users and citizens on AMR through new information and communication technologies (e.g. social media); open-data initiatives, tracking systems, digital and mobile approaches to strengthen health literacy.
	Preparedness	Anticipatory governance	Finding solutions for a better future adaptation	Development of simulation models including feedback loops. Creation of new forecasting tools for anticipatory governance, shared for all actors and fields to deal with emergent events.

Abbreviations: *AMR* antimicrobial resistance, *HCPs* healthcare professionals.

^a^Complexity theory: sense that everything is indeed related to everything else

^b^Inter-dependence: refers to situations characterized by reciprocal effects among countries or among actors in different countries. Interdependence exists where transactions have reciprocal – and not necessarily symmetrical – costly effects

^c^Co-production: achieving outcomes by working together with the involvement and cooperation of citizens and enabled by the proliferation

of new technologies and access to information

^d^Diffusion of governance: whole-of-government and whole-of-society approaches crossing the boundaries of organizations, creating network-based public service production systems

^e^Shared value: policies and operating practices that enhance the competitiveness of a company while simultaneously advancing economic and social conditions in the communities in which it operates

^f^Nudge Policies: interventions based on behavioural science, political theory and economics which propose positive reinforcement and indirect suggestions to try to achieve non-forced compliance to influence the motives, incentives and decision making of groups and individuals

^g^Cybernetic approach: emphasis on central government use of information for guidance and control though feedback as a means of inducing self-correcting behavior at the organizational level

### Participatory governance through empowerment and involvement of healthcare users

A recent meeting of the European Union (EU) Council recognised healthcare users’ empowerment and involvement as an essential part of good quality and safety of care [56]. Patients are both consumers choosing providers of services, and citizens claiming empowerment and participation in governance [57]. Success requires increased participatory governance with empowerment and cooperation of HCPs and patients. Transparency is crucial to gain citizens’ trust and collaboration. The availability of accurate and real-time information may, for example, help patients to understand choices for antibiotics prescribing (according to clinical signs and epidemiology) and IPC measures [58]. Tools such as smartphones and social media can empower people by increasing access to information and facilitate multi-way communication and engagement [59].

In such a participatory process, citizens’ views and preferences would be sought, debated and incorporated into service provision recommendations. In a global matter such as AMR, guideline development groups often include patient’s representatives [60]. Through this process, HCPs and the public are peers who jointly share the responsibility and accountability of authoritative decision making. With their emphasis on cooperation rather than managerial instructions, participatory approaches are more likely to diffuse legitimated policies throughout society and thus promote sustainability.

### Governance mixing hierarchy and network organizations

Successful AMR control strategies require a multi-faceted approach in which all relevant sectors of the healthcare system are engaged. In 2002, the EU recommended to restrict systemic antibacterial agents to prescription-only [61]. Some states relied on multi-lateral organizations to help coordinate policy responses. Previous lack of such top-down governance has contributed to a high proportion of antibiotics sold over the counter [62]. Both top-down and horizontal governance are sometimes purposefully employed together in complex multi-level organizations. A softer side of top-down authority has emerged via multi-level governance to promote self-regulation and growing interest in what are referred to as ‘nudge’ policies [63]. Such policies, based on behavioural science, political theory and economics propose positive reinforcement and rather modest indirect suggestions and triggers to try to achieve non-forced compliance and to influence the motives, incentives and decision making of groups and individuals. In short, to make the safer choice the easier choice [64] but not to command directly.

### The rise of independent agencies and expert bodies

Participatory mechanisms can also be developed through independent agencies, outside of government, but with the capacity and remit to advise ministers through the provision of evidence synthesis and scenario evaluation. Examples include PHE in England and PHF in France, charged with priority setting at the national level. These agencies have responded to the increased demand for information, the need for scientific and expert advice and the need to facilitate new distributions of power. Open consultations and the inclusion of professional and patient associations in an advisory capacity are important mechanisms for distributing knowledge and power more widely. This agency approach represents a new mode of national and international governance, making processes such as risk assessment for health protection arguably more open, objective and less political [65].

### Adaptive and anticipatory governance

Surprise, instability and extraordinary changes are and will be regular features of healthcare delivery. The emergence of resistant organisms is constant. The control of communicable diseases requires flexible and adaptable system approaches. Self-organization and decentralized decision-making can potentially allow the management of major societal risk [66] (as in the case of AMR), more efficiently through ensuring flexibility and the ability to respond to unanticipated challenges. Simulation combined with feedback loops and monitoring can possibly be powerful tools for illustrating possible future complex, multi-actor issues and anticipating emerging situations [67]. Central government’s use of information for guidance and control though feedback as a means of inducing self-correcting behaviour at the organizational level (the cybernetic approach) could support such adaptive management [68].

## Conclusion and policy recommendations

We suggest that the characteristics of the English health policy system generally, and also AMR policy field specifically, diverge from those found in France and Germany in some important ways. It is more top down, more managerial and with a strong emphasis on the setting and monitoring of targets and key performance indicators, and the provision of performance data. An implication is that it will not be easy to align governance systems for AMR across the three countries as they reflect the distinctive health policy fields found in each jurisdiction.

In each of the three countries analysed, key independent organisations which deliver a public service, but that are not ministerial government departments (referred to as arm’s length agencies), are now strongly embedded and accepted within the healthcare infrastructure. The roles of these agencies include priority setting, performance monitoring and are hence a part of the accountability framework. However, this triad of roles require better alignment and also room for temporal and local adaptability. This includes: (i) consistent and clear priorities at the national and local level, with autonomy at the local level (ii) the implementation of meaningful and feasible measures to improve performance which are not counter to other quality improvement objectives and (iii) appropriate mix of incentives and penalties. We observed a variation in setting priorities nationally in France/England and at both national and federal levels in Germany. Countries are also at different stages of performance monitoring; with transparency through public reporting of detailed indicators at opposite ends of the spectrum with an ever-widening scope in England versus confidential reporting (to date) in Germany. Finally, accountability mechanisms appear to vary considerably between countries. The optimal approach is difficult to design and requires a mixed approach given the complexities of individual and organisational behaviours. The effective implementation of governance approaches may well require the extensive involvement of various actors from the national to the front-line level and a level of engagement with the issue of AMR by the public; engagement strategies with the latter are currently not well understood. Agencies need to be mindful that easily accessible and clear information is a prerequisite for the adoption of new mechanisms of governance.

In this way, leaders do not dictate rules and monitor compliance but help stakeholders to achieve shared goals to maintain effectiveness of antimicrobials, mixing regulation and persuasion. Network governance approaches are in our view more likely to be legitimate and sustainable than top down control.

## Abbreviations

AMR: Antimicrobial resistance
DH: Department of Health
ESPAUR: English surveillance programme for antimicrobial utilisation and resistance
G-BA: Federal Joint Committee
GRE: glycopeptide resistant enterococci
HAS: Haute Autorité de Santé
HCP: Healthcare professionals
IfSG: Protection Against Infection Act
IPC: Infection prevention and control
IQTIG: Improvement and Transparency in Health Care
MDRO: Multidrug-resistant organisms
MoH: Ministry of health
MRSA: Methicillin resistant *Staphylococcus aureus*
NHS: National Health Service
PHE: Public Health England
PHF: Public Health France
